# Association of chronic Chagas cardiomyopathy stages with obstructive sleep apnea and excessive daytime sleepiness: a cross-sectional study

**DOI:** 10.3389/fmed.2025.1652618

**Published:** 2025-10-28

**Authors:** Luana F. Cruz, Liliane F. A. Oliveira, Isabela Guimarães Pache de Faria, Vinicius C. Viana, Bruno M. F. Silva, Caroline P. Serrano, Lucia R. N. B. Paes, Mauro F. F. Mediano, Alejandro M. Hasslocher-Moreno, Cláudia Maria Valete

**Affiliations:** ^1^Evandro Chagas National Institute of Infectious Diseases, Oswaldo Cruz Foundation, Rio de Janeiro, Brazil; ^2^Gaffrée e Guinle University Hospital (Unirio), Rio de Janeiro, Brazil; ^3^Department of Otorhinolaryngology, Faculty of Medicine, Federal University of Rio de Janeiro (UFRJ), Rio de Janeiro, Brazil

**Keywords:** Chagas disease, sleep apnea, STOP-Bang questionnaire, Epworth Sleepiness Scale, Chagas cardiomyopathy

## Abstract

**Introduction:**

Chagas disease (CD) remains a significant cause of morbidity and mortality in Latin America. Chronic Chagas cardiomyopathy (CCC) is the most severe clinical form. Obstructive sleep apnea (OSA), a highly prevalent sleep-related breathing disorder, is associated with adverse cardiovascular and metabolic outcomes, yet its clinical relevance in CD remains poorly understood. This study aimed to examine the association between the stages of CCC with the OSA risk and excessive daytime sleepiness (EDS) in patients with CD.

**Methods:**

This cross-sectional observational study included patients with chronic CD. OSA risk was assessed using the STOP-BANG questionnaire; EDS was measured by the Epworth Sleepiness Scale. CCC staging followed the 2nd Brazilian Consensus on CD. Categorical variables were compared using Pearson’s chi-square test and continuous variables with ANOVA. Logistic regression models were fitted to explore associations between CCC stages with OSA risk and EDS.

**Results:**

A total of 133 patients (35.3% men; mean age 67.1 years) were included. Of these, 34.6% had no CCC, 49.6% were CCC stages A/B1, and 15.8% were stages B2/C. Of the 130 participants who completed the STOP-Bang questionnaire, 19.2% were at low risk for OSA (score ≤2), while 80.8% were at intermediate or high risk (score >2). EDS (Epworth Sleepiness Scale >10) was identified in 19.6% of participants. No significant associations were found between CCC stages with either OSA risk or EDS.

**Conclusion:**

Despite a high prevalence of OSA risk among patients with CD, no association was observed between CCC severity and OSA risk or EDS.

## Introduction

Chagas disease (CD) is an infectious disease caused by the protozoan *Trypanosoma cruzi*, affecting approximately 6–8 million people worldwide. Although it remains most prevalent in Latin America, the number of cases has been increasing in non-endemic regions such as North America and Europe due to population mobility. Currently, approximately 70 million people worldwide live in areas considered at high risk for CD (1).

CD can be transmitted through multiple routes. Historically, the primary mode of transmission has been vectorial, but other important pathways include blood transfusion, organ transplantation, congenital transmission, laboratory or occupational exposure, and ingestion of contaminated food or beverages (2). Following infection, the disease typically progresses through an acute phase, often asymptomatic or characterized by mild, nonspecific symptoms, and subsequently into a chronic phase that may persist for decades. The chronic phase manifests in four clinical forms: indeterminate, cardiac, digestive, and mixed. Among the chronic forms of CD, the indeterminate form is the most prevalent clinical presentation, accounting for approximately 50–70% of cases. Indeterminate form is characterized by the presence of anti-*Trypanosoma cruzi* antibodies in the absence of specific clinical, electrocardiographic, or radiological abnormalities attributable to the disease. This clinical form is generally considered benign, with a low risk of progression in the short and medium term (3). Determinate forms of CD include the digestive and cardiac forms, the latter known as chronic Chagas cardiomyopathy (CCC), which accounts for approximately 30% of all cases and is responsible for the high morbidity and mortality. The main manifestations of CCC include heart failure, arrhythmias, atrioventricular blocks, and thromboembolism (4). Additionally, patients are classified according to the severity of cardiac involvement, as determined by electrocardiographic and echocardiographic abnormalities, following the criteria established by the 2nd Brazilian Consensus on Chagas Disease (5).

Obstructive sleep apnea (OSA) is a highly prevalent sleep-related breathing disorder, characterized by recurrent episodes of total or partial upper airway obstruction during sleep, affecting approximately 22% of men and 17% of women (6). OSA can be influenced by genetic, anatomical, hormonal, and neuromuscular factors, being considered a significant public health concern due to its strong association with an increased risk of cardiovascular and metabolic disorders (7). Moreover, a commonly reported symptom of OSA is excessive daytime sleepiness (EDS), which results from fragmented and poor-quality sleep, and has been associated with reduced quality of life and increased mortality (8).

The coexistence of CD and OSA may worsen the patient’s clinical condition, leading to a poorer prognosis, increased need for medical interventions, and a reduced quality of life. Despite limited data, a previous study reported a high prevalence of OSA in individuals with CD and suggested a potential association of the presence and severity of CCC with OSA (9). To address this gap in literature, this study aimed to examine the association between the stages of CCC (exposures) with the risk of OSA and EDS (outcomes) in patients with CD.

## Methods

### Study design and settings

This cross-sectional observational study included patients with CD regularly followed at the Laboratory of Clinical Research on Chagas Disease (Lapclin-Chagas) of the Evandro Chagas National Institute of Infectious Disease (INI) of the Oswaldo Cruz Foundation (Fiocruz) in Rio de Janeiro, Brazil. Data was collected from September 2022 to August 2023. Inclusion criteria were: (1) age ≥18 years and (2) positive IgG anti-*Trypanosoma cruzi* detected through two serological tests with different principles or different antigenic preparations. Patients were excluded if they: (1) were unable to complete the questionnaires without assistance; or had (2) neurodegenerative diseases, (3) decompensated heart failure, (4) decompensated chronic kidney disease, or (5) associated infectious diseases.

### Exposure variable

The exposure assessed in this study was the severity of CCC. The staging of CCC was based on the criteria established by the 2nd Brazilian Consensus on Chagas Disease (5), as follows: A – abnormal electrocardiogram (ECG) with a normal echocardiogram (ECHO); B1 – abnormal ECG with abnormal ECHO showing left ventricular (LV) wall motion abnormalities and an LV ejection fraction (LVEF) ≥ 45%; B2 – abnormal ECG with abnormal ECHO showing LV wall motion abnormalities and LVEF < 45%; C – abnormal ECG and ECHO with compensated heart failure; and D – abnormal ECG and ECHO with refractory heart failure.

### Outcome variables

The outcome variables considered in this study were the risk of OSA and EDS. The risk of OSA was assessed using the STOP-Bang Questionnaire (SBQ), which consists of eight dichotomous (yes/no) items, scored as 1 or 0, respectively, for a total score ranging from 0 to 8. The SBQ has been translated, culturally adapted, and validated for use in the Brazilian population (10). Based on the total SBQ score, individuals were classified as being at low (0–2), intermediate (3–4), or high (>4) risk for OSA. EDS was assessed using the Epworth Sleepiness Scale (ESS), a self-reported questionnaire also translated and validated in Brazil. The ESS evaluates the likelihood of falling asleep in eight daily situations, with each item scored from 0 to 3, resulting in a total score ranging from 0 to 24. Scores higher than 10 indicate excessive daytime sleepiness (11).

### Covariates

Sociodemographic and clinical data were extracted from participants’ electronic medical records to describe the study sample and to adjust for potential confounding variables. Race was categorized as white or non-white (including black, mixed, and indigenous). Recorded comorbidities comprised hypertension, diabetes, dyslipidemia, and pulmonary disease. Additionally, participants were asked about prior polysomnography, previous diagnosis of OSA, and previous or current use of continuous positive airway pressure (CPAP). The classification of the digestive forms of CD was based on the presence of symptoms consistent with megacolon or megaesophagus, in accordance with the criteria established by the 2nd Brazilian Consensus on Chagas Disease.

### Data analysis

Statistical analysis was performed using the Statistical Package for Social Sciences for Windows version 16.0 (SPSS Inc., Chicago, IL, USA). The REDCap (Research Electronic Data Capture) platform was used for data management. Frequencies and percentages of categorical variables, as well as means and standard deviations of continuous variables, were described. The normality of continuous variables was verified using the Shapiro–Wilk and Kolmogorov–Smirnov tests. Categorical variables were compared using Pearson’s chi-square test, while mean values of continuous variables were compared using analysis of variance (ANOVA).

The association between the stages of CCC and the risk of OSA, classified as intermediate and high risk (SBQ > 2) or high risk (SBQ > 4), as well as EDS (ESS > 10), were evaluated using logistic regression models, with all outcomes treated as dichotomous categorical variables. Unadjusted and adjusted models were fitted considering the following potential confounding variables: age, sex, number of comorbidities (0, 1, ≥2), and digestive form of CD. A statistical significance level of *p* ≤ 0.05 was adopted for all tests.

## Results

The study included 133 patients with a mean age of 67.1 years (±10.9), of whom 35.3% were men. Regarding race, 39.9% self-identified as white. Hypertension was the most prevalent comorbidity (82.7%), followed by dyslipidemia (55.6%) and diabetes mellitus (30.1%). The study sample comprised 34.6% of patients with no CCC, 49.6% with CCC classified as stages A and B1, and 15.8% with CCC as stages B2 and C. Digestive form of CD was presented in 24.8% of participants. A minority of participants reported prior polysomnography (1.5%), and none had a previous diagnosis of OSA. Only one participant (0.8%) reported previous/current use of CPAP. Among the 130 participants who completed the SBQ (3 missing SBQ due to report error), 19.2% were classified as low risk for OSA (score ≤2), while 80.8% were at intermediate or high risk (score >2). EDS (ESS > 10) was identified in 19.6% of participants.

The comparison between groups classified according to the stages of chronic CCC revealed that mean age increased significantly with disease severity (*p* < 0.001). A significantly higher proportion of men was observed in the more advanced stages, 57.1% in stages B2 + C versus 23.9% in the no CCC group (*p* = 0.03). Hypertension was also more prevalent among those with more severe cardiac involvement, reaching 100% in the B2 + C group (*p* < 0.001). The digestive form of CD was more common in the A + B1 group (33.3%) and showed a borderline association across CCC stages (*p* = 0.05). Other characteristics, such as diabetes, dyslipidemia, pulmonary disease, excessive daytime sleepiness, and risk of OSA, did not differ significantly between the groups. These findings are summarized in Table 1.

**Table 1:** Characteristics of participants (overall and stratified by stages of CCC).

		Total (*n* = 133)	No CCC (34.6%, 46)	Stages A + B1 (49.6%, 66)	Stages B2 + C (15.8%, 21)	*p*-value
Age in years (mean, sd)		67.1 (±10.9)	62.2 (±9.6)	70.7 (±10.4)	66.8 (±11.7)	**<0.001**
Men (%, *n*)		35.3%, 47	23.9%; 11	36.4%; 24	57.1%; 12	**0.03**
White (vs. non-white)		39.9%, 53	45.7%, 21	37.9%, 25	33.3%, 7	0.57
Digestive form (%, *n*)		24.8%, 33	13.0%; 6	33.3%, 22	23.8%, 5	**0.05**
Hypertension (%, *n*)		82.7%, 110	63.0%, 29	90.9%, 60	100%, 21	**<0.001**
Diabetes mellitus (%, *n*)		30.1%, 40	23.9%, 11	34.9%, 23	28.6%, 6	0.46
Dyslipidemia (%, *n*)		55.6%, 74	52.2%, 24	57.6%, 38	57.1%, 12	0.84
Pulmonary disease (%, *n*)		3.8%, 5	2.2%, 1	4.5%, 3	4.8%, 1	0.78
Number of comorbidities (%; *n*)	0	7.5%, 10	15.2%, 7	4.6%, 3	0.0%, 0	0.06
	1	33.1%, 44	39.1%, 18	28.8%, 19	33.3%, 7	
	2+	59.4%, 79	45.7%, 21	66.7%, 44	66.7%, 14	
Prior polysomnography (%, *n*)		1.5%, 2	2.2%, 1	1.5%, 1	0.0%, 0	0.79
Previous diagnosis of OSA (%, *n*)		0.0%, 0	0.0%, 0	0.0%, 0	0.0%, 0	NR
Previous/current use of continuous positive airway pressure (%, *n*)		0.8%, 1	2.2%, 1	0.0%, 0	0.0%, 0	0.39
Low risk of OSA (Stop Bang ≤2)		19.2%, 25	26.1%, 12	14.3%, 9	19.1%, 4	0.30
Intermediate and high risk of OSA (Stop Bang >2) (*n* = 130)		80.8%, 105	73.9%, 34	85.7%, 54	80.9%, 17	
Excessive daytime sleepiness (ESS ˃ 10)		19.6%, 26	17.4%, 8	21.2%, 14	19.1%, 4	0.88

NR, Not reported due to insufficient cases for statistical comparisons. Estimates in bold are statistically significant.

The association between the stages of CCC with both the risk of OSA and EDS is presented in Table 2. In the unadjusted models, no statistically significant associations were observed between CCC stages and OSA risk using either the STOP-Bang cutoff points of >2 or >4, nor with EDS (ESS > 10). Similarly, adjusted models (considering age, sex, associated digestive form, and number of comorbidities) did not show any statistically significant association (Table 2).

**Table 2:** Association between the stages of CCC with risk of OSA (SBQ) and excessive daytime sleepiness (ESS).

	Unadjusted	Adjusted*
SBQ > 2 (*n* = 105)	OR (CI 95%)	
No CCC	Reference	Reference
Stages A + B1	2.12 (0.81–5.56)	1.12 (0.36–3.49)
Stages B2 + C	1.50 (0.42–5.36)	0.93 (0.21–4.22)
SBQ > 4 (*n* = 38)	OR (CI 95%)	
No CCC	Reference	Reference
Stages A + B1	1.22 (0.52–2.86)	0.89 (0.33–2.41)
Stages B2 + C	1.42 (0.46–4.35)	1.01 (0.29–3.53)
ESS > 10 (*n* = 26)	OR (CI 95%)	
No CCC	Reference	Reference
Stages A + B1	1.28 (0.49–3.35)	1.24 (0.42–3.61)
Stages B2 + C	1.12 (0.30–4.22)	1.01 (0.25–4.15)

*Adjusted models included the following potential confounders: age, sex, number of comorbidities, and the digestive form of CD.

## Discussion

In this cross-sectional study of patients with CD, we identified a high prevalence of individuals at intermediate to high risk for OSA, while the prevalence of EDS was relatively low. However, no significant associations were observed between CCC stages and either OSA risk or EDS. OSA remains substantially underdiagnosed, with an estimated 80% of men and 93% of women with the condition going undetected (12). While a previous study including 287 patients with CD did not find any participant with a previous diagnosis of OSA (9), only one patient in our study reported having been diagnosed with the condition. Despite this, most participants in our study were classified as being at intermediate or high risk for OSA. This prevalence is higher than reported in other studies, which identified 58% at intermediate and 12% at high risk for OSA (8). The higher prevalence of OSA in our study was expected, as we used a screening questionnaire, while others employed polysomnography, the diagnostic gold standard.

The apparent discrepancy between the high prevalence of individuals at intermediate to high risk for OSA and the relatively low prevalence of EDS may be explained by some factors. The ESS has recognized limitations in sensitivity and relies on self-reported data, which may underestimate EDS in specific populations and be influenced by reporting bias, particularly among individuals with chronic diseases (13, 14). Moreover, patients with CD may experience physiological adaptation to long-standing sleep-disordered breathing, reducing their perception of daytime sleepiness (15). These findings suggest that relying solely on subjective measures of EDS may underestimate the clinical impact of OSA in this population and highlight the need for objective assessments when evaluating sleep-related symptoms in patients with CD.

In our study, we did not find an association between the clinical stages of CCC and the risk of OSA. This contrasts with a previous study that reported a higher prevalence of ventricular dysfunction in patients with moderate to severe OSA compared to those with mild or no OSA (9). Several factors may have contributed to this discrepancy. First, our study relied on a screening questionnaire rather than polysomnography, the gold standard for diagnosing OSA, which may have attenuated associations and limited our ability to detect true relationships due to increased measurement error. Second, our sample included a relatively small number of patients in the more advanced CCC stages (stages B2 and C), which likely reduced statistical power and may have limited our ability to detect statistically significant associations. Third, disease-specific mechanisms in CD may differ from those in other cardiomyopathies: autonomic dysfunction, prevalent in CD, could modulate ventilatory control and OSA expression in ways that remain poorly understood.

The high proportion of individuals at risk for OSA in our sample highlights the need to integrate systematic screening into the routine care of patients with CD, regardless of CCC stage. The SBQ may serve as a pragmatic tool to identify candidates for definitive diagnostic testing, such as polysomnography. Although evidence regarding the management of OSA specifically in CD remains limited, it is reasonable to extrapolate from broader clinical practice and offer appropriate treatment. In this context, CPAP represents a first-line option for patients with moderate to severe OSA (16), given its favorable safety profile and well-documented effects on sleep quality (17). By improving oxygenation and minimizing sleep fragmentation, CPAP may enhance daily functioning, improve EDS, and increase neuropsychological performance and overall quality of life (18). Even in the absence of a clear association with CCC severity, untreated OSA may contribute to poor sleep quality, impaired quality of life, and adverse cardiovascular events (15, 16). Furthermore, as CCC progression and OSA may share pathophysiological pathways, future research should prioritize evaluating whether treating sleep-disordered breathing can influence the natural history of CCC.

Several limitations should be acknowledged from the present study. First, the cross-sectional design precludes causal inferences. Second, the use of a screening questionnaire rather than polysomnography—the gold standard for diagnosing OSA—may have introduced misclassification bias, which reduces the validity of the risk estimates and weakened the observed associations, particularly in differentiating true OSA from other causes of EDS. Third, our sample included a relatively small number of patients in the more advanced CCC stages (B2 and C), which may have limited statistical power and increased the risk of type II error, potentially underestimating true associations. Fourth, we did not account for other potential causes of EDS, such as depression, sedative medication use, or other sleep disorders, which may have introduced residual confounding and affected the interpretation of our results. Finally, our sample may not fully represent the broader CD population, as it was drawn from a single reference center.

In conclusion, we observed a high prevalence of individuals at risk for OSA among patients with CD, independent of CCC severity. No statistically significant association was observed between CCC severity and OSA risk or EDS. These findings suggest that clinicians should consider systematic OSA screening in CD patients regardless of CCC stage, as undetected OSA may still adversely impact cardiovascular outcomes. Future studies employing objective sleep assessment and longitudinal designs are needed to clarify the clinical impact of OSA in CD and its potential interactions with cardiac disease progression.

## Data Availability

The raw data supporting the conclusions of this article will be made available by the authors, without undue reservation.
